# Polyphenols with Anti-Proliferative Activities from *Penthorum Chinense* Pursh

**DOI:** 10.3390/molecules190811045

**Published:** 2014-07-29

**Authors:** Doudou Huang, Yun Jiang, Wansheng Chen, Fengyan Yao, Lianna Sun

**Affiliations:** 1 Department of Identification of Traditional Chinese Medicine, School of Pharmacy, Second Military Medical University, Shanghai 200433, China; E-Mails: hdd890920@163.com (D.H.); yaofy2013@163.com (F.Y.); 2 State Key Laboratory of Quality Research in Chinese Medicine, Institute of Chinese Medical Science, University of Macau, Macau, China; E-Mail: jiangyun@163.com; 3 Department of Pharmacy, Changzheng Hospital, Shanghai 200003, China; E-Mail: chenws126@126.com

**Keywords:** *Penthorum chinense*, polyphenols, anti-proliferative activity

## Abstract

Two new polyphenols, penthorumin C (**1**) and 2,6-dihydroxyacetophenone-4-*O*-[4ꞌ,6ꞌ-(S)-hexahydroxydiphenoyl]-β-d-glucose (**2**), along with four known polyphenolic acids, pinocembrin-7-*O*-[4ꞌꞌ,6ꞌꞌ-hexahydroxydiphenoyl]-β-d-glucose(**3**), pinocembrin-7-*O*-[3ꞌꞌ-O-galloyl- 4ꞌꞌ,6ꞌꞌ-hexahydroxydiphenoyl]-β-d-glucose (**4**), thonningianin A (**5**), and thonningianin B (**6**) were isolated from *Penthourm chinense*. All compounds were evaluated for their anti-proliferative activity in HSC-T6 cells, and **2** and **5** showed significant activity, with IC_50_ values of 12.7 and 19.2 μM, respectively.

## 1. Introduction

Oxidative stress due to the imbalance in oxidant and antioxidant status has been implicated in the pathogenesis of several diseases, including cancer. Several papers have reported the role of free radical mediated oxidative damage in multi-stage events of carcinogenesis. Polyphenolic acids are a complex group of compounds that have attracted attention in the last few years because of their widespread occurrence on plants and their significant biological activities [1,2]. Polyphenols may have therapeutic health effects for a variety of chronic pathological conditions including cancer, neurodegenerative diseases, diabetes, and cardiovascular diseases due to the antioxidant activity. Previous reports have showed that polyphenolic acids are the inhibition of hepatic stellate cells proliferation, considering to be associated to liver fibrosis [3]. 

**Figure 1:**
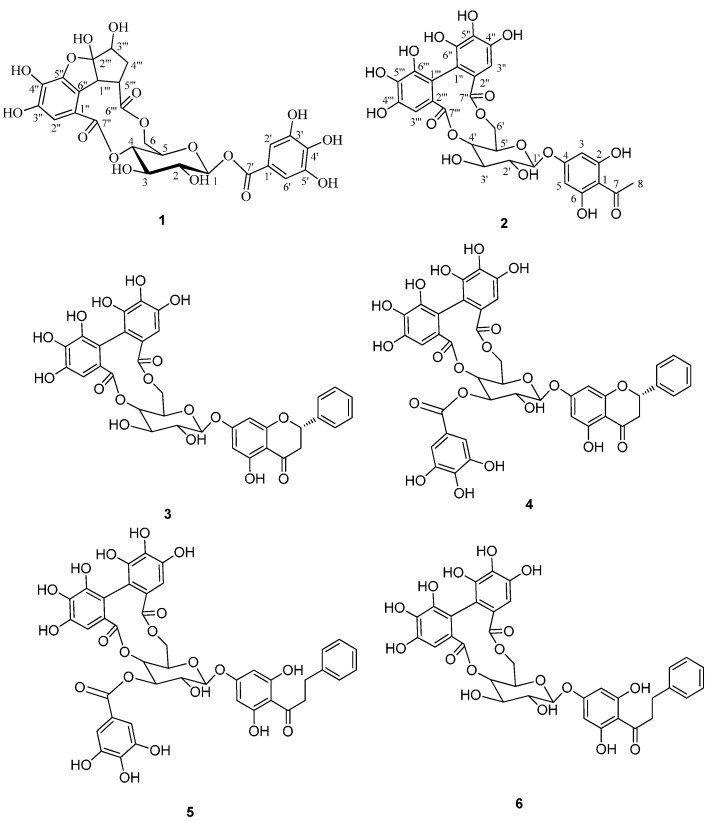
Compounds **1**–**6** isolated from *Penthourm chinense.*

*Penthorum chinense* Pursh, (Saxifragaceae), is widely distributed in eastern Asia, (China, Japan, Korea, and eastern Russia) [4]. In China, where it is both consumed as food and used in traditional Chinese medicine, whole plant products prepared from *P. chinense* is used to alleviate “heat”-associated disorders, detoxification, to promote circulation, and to treat liver problems, and to protect the spleen [5]. *P. chinense* metabolites have shown anti-oxidant and antitumor activities [6,7,8,9]. Flavonoids, triterpenoids, polyphenols, and lignans have been isolated from *P. chinense* [10]. Two new polyphenols (compounds **1** and **2**), along with four polyphenols (compounds **3**–**6**) were isolated from the aerial parts of *P. chinense* (Figure 1). Herein, we describe the separation and structural characterization of these compounds, as well as their anti-proliferative activity in HSC-T6 cells induced by PDGF.

## 2. Results and Discussion

### 2.1. Structure Elucidation

An 80% ethanolic extract of dried *P. chinense* whole plant was suspended in distilled water and extract with petroleum ether, EtOAc and *n*-BuOH. The EtOAc soluble fraction was concentrated under reduced pressure to produce a residue that was subjected multiple chromatography, two new compounds **1** and **2** and four known compounds were isolated and identified.

**Figure 2:**
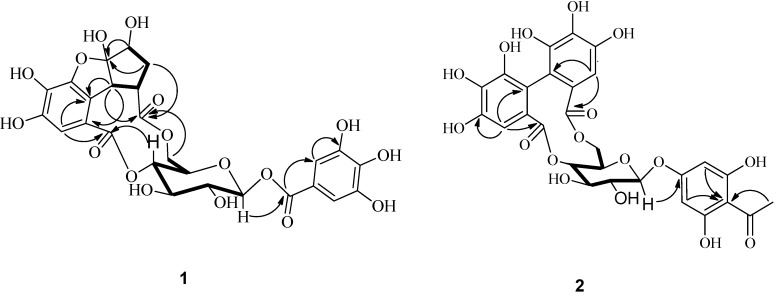
^1^H-^1^H COSY (▬) and key HMBC (**→**) correlations of compounds **1** and **2**.

Compound **1** was obtained as white powder. The molecular formula C_26_H_24_O_17_ was deduced from the HERSIMS ion peak at *m/z* 607.0948 [M−H]^−^ (calcd *m/z* 607.0901), exhibiting fifteen degrees of hydrogen deficiency. Its IR spectrum contained characteristic absorptions for hydroxyl (3407.6 cm^−1^), carbonyl (1724.0 cm^−1^) and aromatic ring (1619.9, 1454.0 cm^−1^) moieties. Interpretation of the NMR spectra (Table 1) suggested the presence of a glucose (C-1 to C-6), and two galloyl (C-1ꞌ to C-7ꞌ; C-1ꞌꞌ to C-7ꞌꞌ) groups (Ikuko *et al*., 2000). The glucopyranose moiety was determined to have a *β*-configuration at C-1 with the large coupling constant of H-1 (*J* = 7.8 Hz). The remaining six carbons were assigned as one carbonyl (δ_C_ 173.6), one methylene (δ_C_ 35.0), two methines (δ_C_ 55.7, 75.4) including one (δ_C_ 75.4) connected to an oxygen atom, and one doubly oxygen-substituted quaternary carbon (δ_C_ 116.8). The HMBC spectrum (Figure 2) exhibited long-range correlations from H_2_-4ꞌꞌꞌ to C-1ꞌꞌꞌ, C-2ꞌꞌꞌ, C-3ꞌꞌꞌ, and C-5ꞌꞌꞌ, consistent with a cyclopentyl ring. The presence of a 1,4,6-tri-*O*-substituted-*β*-glucopyranose was deduced from HMBC correlations from H-4 to C-7ꞌꞌ, from H-1 to C-7ꞌ, and from H_2_-6 to C-6ꞌꞌ. The HMBC correlations from H-1ꞌꞌꞌ to C-1ꞌꞌ, C-5ꞌꞌ, C-6ꞌꞌ and from H-1ꞌꞌꞌ, H-4ꞌꞌꞌ, H-5ꞌꞌꞌ to C-6ꞌꞌꞌ indicated that the cyclopentyl ring was connected to C-6ꞌꞌ and C-6ꞌꞌꞌ via C-1ꞌꞌꞌ/C-6ꞌꞌ and C-5ꞌꞌ/C-6ꞌꞌꞌ. The glucopyranose, substituted galloyl moieties, and an additional ring account for 15 degrees of hydrogen deficiency. On the basis of the molecular formula and the chemical shift of C-2ꞌꞌꞌ (δ_C_ 116.8), the cyclopentyl ring was also connected to C-5ꞌꞌ via oxygen linkage to form the remaining ring [11]. The absolute configuration of glucose was proposed to be D by comparison of its specific rotation, [α]_D_ −21° (*c* 0.16, MeOH), with those reported for related compounds [12,13].

**Table 1:** ^1^H (600MHz) and^13^C (150 MHz) NMR spectroscopic data of compound **1** in DMSO-*d*_6_.

Position	δ H	δ C
1	5.66(1H, d, 7.8)	94.4
2	3.40(1H, t, 9.0)	73.0
3	3.58(1H, t, 9.0)	73.2
4	5.07(1H, t, 9.6)	74.5
5	4.07(1H, t, 4.2)	66.8
6	4.49(1H, dd, 3.6, 10.8)3.92(1H, d, 4.2)	64.6
1ꞌ	-	118.4
2ꞌ, 6ꞌ	7.03(2H, s)	109.2
3ꞌ, 5ꞌ	-	145.7
4ꞌ	-	139.4
7ꞌ	-	164.7
1ꞌꞌ	-	117.7
2ꞌꞌ	6.82(1H, s)	110.6
3ꞌꞌ	-	146.1
4ꞌꞌ	-	133.6
5ꞌꞌ	-	146.8
6ꞌꞌ	-	119.7
7ꞌꞌ	-	167.1
1ꞌꞌꞌ	3.68(1H, d, 9.0)	55.7
2ꞌꞌꞌ	-	116.8
3ꞌꞌꞌ	3.96(1H, dd, 6.6, 12.0)	75.4
4ꞌꞌꞌ	2.04(1H, d, 6.0)1.93(1H, d, 12.0)	35.0
5ꞌꞌꞌ	2.42(1H, d, 6.0)	45.3
6ꞌꞌꞌ	-	173.6

**Figure 3:**
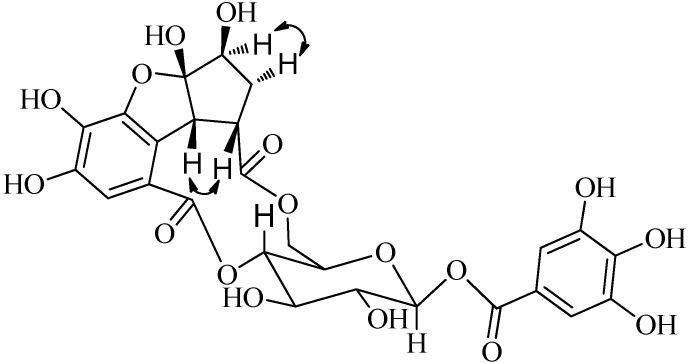
Selected NOESY correlations (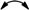) of compound **1**.

The relative configuration of **1** was confirmed by observance of NOESY correlations (Figure 3), that were observed between H-3ꞌꞌꞌ to H-4ꞌꞌꞌ α, and H-5ꞌꞌꞌ, H-1ꞌꞌꞌ to H-4ꞌꞌꞌ β. These correlations suggested that H-3ꞌꞌꞌ was α-oriented and both H-5ꞌꞌꞌ and H-1ꞌꞌꞌ were both β-orientated. To minimize the energy of **1** the OH-2ꞌꞌꞌ was assigned as β-orientation. The structure of compound **1** (penthorumin C) was thus assigned as shown in Figure 1. 

Compound **2** was obtained as white powder with a molecular formula of C_28_H_24_O_17_ according to HRESIMS (*m/z* [M+H]^+^ 633.1088, calcd *m/z* 633.1019) constant with 17 degrees of hydrogen deficiency. The IR spectrum indicated hydroxyl (3407.6 cm^−1^), carbonyl (1731.7 cm^−1^) and aromatic ring (1627.6, 1596.7 cm^−1^) moieties were present. The ^1^H-NMR showed resonances for one methyl group (δ_H_ 2.61, 3H, s). The ^13^C-NMR data (Table 2) exhibited resonances for four methines at δ_C_ 71.7 (C-4ꞌ), 73.8 (C-2ꞌ), 73.8 (C-3ꞌ) and 71.1 (C-5ꞌ), one methylene at δ_C_ 62.9 (C-6ꞌ) and one anomeric methine at δ_C_ 99.8 (C-1ꞌ), indicating the presence of a glucopyranose unit. The glucopyranose moiety was determined to have a C-1ꞌ β-orientation by observance of H-1ꞌ (*J* = 7.8 Hz) coupling constant. The ^1^H and ^13^C-NMR spectra of **2** (Table 2) are similar to those of thonningianin B [14], except for the absence of the resonances corresponding to one benzyl group. The HMBC correlations from H_3_-8 to C-7 and C-1, suggested that the acetyl group (C_7_-C_8_) was connected to C-1. The CD spectrum of **2** shows a negative Cotton effect at 262.5 nm (Δ −5.6) and a positive effect at 241.5 nm (Δ +23.0), indicating an *S*-configuration of the hexahydroxydiphenoyl (HHDP) group [14]. The absolute configuration of glucose was proposed to be D by comparison of its specific rotation, [α]_D_ −31° (*c* 0.21, MeOH), with those reported for related compounds [12]. The structure of compound **2** (2,6-dihydroxyacetophenone-4-*O*-[4',6'-(*S*)-hexahydroxydiphenoyl]-β-_d_-glucose) was thus assigned as shown in Figure 1.

**Table 2:** ^1^H (600MHz) and^13^C (150 MHz) NMR spectroscopic data of compound **2** in DMSO-*d*_6_.

Position	δ H	δ C
1	-	105.8
2, 6	-	164.0
3, 5	6.08(2H, s)	95.0
4	-	163.3
7	-	203.6
8	2.61(3H, s)	32.8
1ꞌ	5.04(1H, d, 7.8)	99.8
2ꞌ	3.57(1H, t, 9.6)	73.8
3ꞌ	3.36(1H, t, 9.0)	73.8
4ꞌ	4.61(1H, t, 9.6)	71.7
5ꞌ	4.13(1H, m)	71.1
6ꞌ	4.98(1H, dd, 6.0, 13.2) 3.74(1H, d, 13.2)	62.9
1ꞌꞌ	-	115.3
2ꞌꞌ	-	124.5
3ꞌꞌ	6.35(1H, s)	105.3
4ꞌꞌ	-	144.3
5''	-	135.1
6''	-	144.6
7ꞌꞌ	-	167.9
1ꞌꞌꞌ	-	115.6
2'''	-	124.8
3'''	6.54(1H, s)	106.3
4'''	-	144.4
5'''	-	135.4
6'''	-	144.6
7'''	-	167.1

Additionally, the known **3**–**6** were identified as pinocembrin-7-*O*-[4ꞌꞌ,6ꞌꞌ-hexahydroxydiphenoyl]-β-d-glucose (**3**) [15], pinocembrin-7-*O*-[3ꞌꞌ-O-galloyl-4ꞌꞌ,6ꞌꞌ-hexahydroxydiphenoyl]-β-d-glucose (**4**) [16], thonningianin A (**5**) [14], and thonningianin B (**6**) [14] by comparison of their physicochemical data with the reported values.

### 2.2. Cytotoxicity of Penthorum Chinense Extract

**Figure 4:**
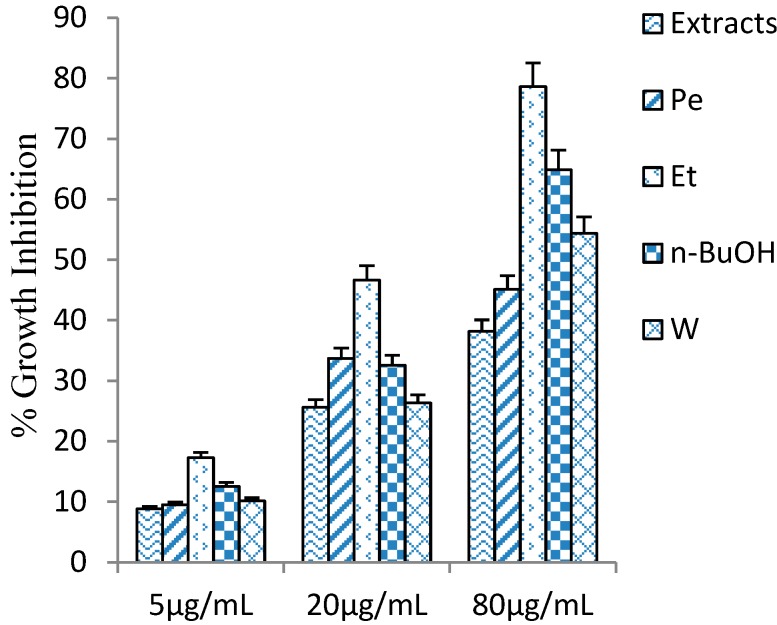
The inhibitory activity of *Penthorum chinense* extracts and four fractional extractions on HSC-T6 cells. Values are mean ± SD, n = 6.

*Penthorum. chinense* extracts were evaluated on the HSC-T6 cells for culture periods of 24 h. *P. chinense* extract and four fractions (Pe, Et, *n*-BuOH, and W), showed little toxic effect under 80 μg/mL (Figure 4). The cytotoxicity of *n*-BuOH fraction was stronger than other fractions (*P. chinense* extract, Pe, Et, and W), and half of the cells were dead when the added concentration was up to 160 μg/mL.

### 2.3. Effect on PDGF Induced Proliferation

Based on the cytotoxic effect on HSC-T6 cells, we evaluated the anti-proliferative activity at 80 μg/mL. In order to figure out the active constituents of *P. chinense*, *P. chinense* extract was suspended in water and partitioned successively with petroleum ether (Pe), ethyl acetate (Et), *n*-BuOH(n-BuOH), and water (W), and these four fractions (Pe, Et, n-BuOH, and W) were tested their inhibitory activity, together with *P. chinense* extract (Figure 4). In comparison with other parts of *P. chinense*, Et fraction showed remarkable inhibitory activity, 78.6% at 80 μg/mL. The result suggested that the active ingredients of anti-proliferation may exhibit in Et part, and some isolation and purification methods had been further applied in Et part, which led to isolation of six polyphenols **1**–**6**, including two new isolates **1** and **2**. The anti-proliferative activities of **1**–**6** were evaluated in parallel experiments by measuring their inhibitory activities in PDGF induced HSC-T6. Compounds **2** and **5** displayed moderate inhibitory activity, with IC_50_ values of 12.7, 19.2 μM, respectively *vs.* 6.36 μM for Colchicine, whereas the other compounds exhibited weak activity, with IC_50_ values greater than 200 μM.

## 3. Experimental

### 3.1. General

Optical rotations were measured on a Perkin-Elmer 341 digital polarimeter at 589 nm. CD spectra were recorded by a JASCO J-810 spectropolarimeter. IR spectra were recorded as KBr disks on an Intelligent Fourier Nicolet FTIR 6,700 Infrared Spectrometer. The NMR spectra, including ^1^H, ^13^C, DEPT and 2D-NMR, were recorded on a Bruker AM-400 and AC-600 spectrometer with chemical shifts reported as δ values, using TMS as internal standard. HRESIMS data were obtained on an Agilent Technologies 6,538 UHD Accurate-Mass Q-TOF LC/MS spectrometer (Agilent Technologies, Waltham, MA, USA). Thin-layer chromatography was performed on TLC plates (Silica gel HSGF254. Jiangyou Company of Yantai, Yantai, China; RP-18 F254. Merck, Darmstadt, Germany) and spots were visualized by heating after dipping into 10% H_2_SO_4_. Silica gel (100–200 and 200–300 mesh, Jiangyou Company of Yantai, Yantai, China), Sephadex LH-20 (Pharmacia, Fairfield, NJ, USA), RP-C18 (43–60 μm, Merck), and MCI gel (Mitsubishi Chemical Corporation, Hongkong, China) were used for column chromatography.

### 3.2. Plant Material

The dry aerial parts of *P. chinense* were provided by Gulin Gansu Pharmaceutical Co., Ltd (Sichuan, China) in September 2009. The plant was identified by Prof. Wansheng Chen. A voucher specimen (No. IT100629) was deposited in the Department of Pharmacognosy of the Second Military Medical University, Shanghai, China.

### 3.3. Extraction and Isolation

The dry aerial parts of *P. chinense* (10 kg) were extracted three times with 80% EtOH (3 × 80 L, 2 h each) at 80 °C, and concentrated under vacuum to give a residue (1,167 g). The residue was suspended in water (1.5 L) and then successively partitioned with petroleum ether (3 × 1.0 L), EtOAc (3 × 2.0 L), and *n*-BuOH (2 × 1.5 L) to give petroleum ether-soluble (99 g), EtOAc-soluble (245 g), and *n*-BuOH-soluble (239 g) extracts. The EtOAc extract (100 g) was separated by chromatography using a silica gel column and eluted with a gradient of CH_2_Cl_2_–CH_3_OH (50:1, 30:1, 10:1, 5:1, 2:1, and 1:1, v/v, each 2 L) to give six fractions (F1–F6). Fraction Fr4 (25.4 g) was divided into five subfractions (Fr-4.1–Fr-4.5) through a silica gel column employing a petroleum ether–acetone gradient (20:1, 10:1, 5:1, 2:1, and 1:1, each 150 mL). Compound **1** (25 mg) and **2** (21 mg) were purified from fraction F4.5 (3.2 g) by Sephadex HL-20 with a gradient of MeOH–H_2_O (50:50, 70:30, each 350 mL). Fraction F4.2 (2.3 g) was applied to ODS column with of MeOH–H_2_O (50:50, 400 mL) to give **3** (16 mg). Compounds **4** (18 mg) and **6** (15 mg) were obtained from fraction F4.3 (7.0 g) which was subjected to ODS column and eluted with MeOH–H_2_O (50:50, 70:30, each 550 mL). Compound **5** (11 mg) was isolated from F4.4 (1.2 g) by reverse-phase HPLC with H_2_O/CH_3_CN 80/20 to 50/50 over 30 min (2 mL/min). The degrees of all compounds were higher than 95% based on TLC and HPLC-DAD-ELSD.

### 3.4. Characterization of Compound **1** and Compound **2**

*Penthorumin C* (**1**). White powder; 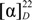 +34.7 (*c* 0.19, CH_3_OH); IR (KBr) *v*_max_ 3407.6, 1724.0, 1619.9, 1454.0, 1214.9, 1033.6, 763.7, 547.7 cm^−1^; ^1^H-NMR and ^13^C-NMR data, see Table 1; HRESIMS *m/z* 631.0901 [M+Na]^+^ (calcd for C_22_H_24_O_17_Na, 631.0906).

*2,6-Dihydroxyacetophenone-4-O-[4ꞌ,6ꞌ-(S)-hexahydroxydiphenoyl]-β-d-glucose* (**2**). White powder; 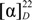 −21.7 (*c* 0.23, CH_3_OH); IR (KBr) *v*_max_ 3407.6, 1731.7, 1627.6, 1596.8, 1517.7, 1442.5, 1363.4, 1286.3, 1232.3, 1174.4, 1018.2, 962.3, 831.2, 742.5, 566.9 cm^−1^; ^1^H-NMR and ^13^C-NMR data, see Table 2; HRESIMS *m/z* 633.1083 [M+H]^+^ (calcd for C_28_H_25_O_17_H, 633.1019).

### 3.5. Cell Culture

An immortalized rat hepatic stellate cell line, HSC-T6 (obtained from the cell bank of the Chinese Academy of Science, Shanghai, China) was were batch cultured in Dulbecco’s Modified Eagle’s Medium (DMEM) with 10% fetal bovine serum (FBS), 100 IU mL^−1^ and streptomycin at 37 °C in a 5% CO_2_ incubator. 

### 3.6. Cell Viability

Compounds and *P. chinense* extract were dissolved in dimethylsulfoxiode (DMSO). Our preliminary study showed that DMSO at a final concentration of 0.1% in media did not affect the cell viability. HSC-T6 cells seeded for 24 h in 96-well plates were exposed to different concentrations of compounds and *P. chinense* extract. Different concentrations of compounds were carried out in the plate for 5 consecutive wells (final volume 100 μL) and incubated for 24 h. Cell viability was calculated as 100 ° (absorbance of treated compound − absorbance of background light)/(absorbance of control − absorbance of background light).

### 3.7. Activation of HSC-T6 Cells Induced by PDGF

HSC-T6 cells were plated in a 96-well plate. Initially, cells were cultured with DMEM containing 10% FBS for 6 h. The medium was then replaced with DMEM without FBS to starve the cells for 12 h. The cells were then cultured with DMEM that contained 5.0 ng/mL PDGF (without FBS) for 24 h.

### 3.8. Anti-Proliferative Activity Assay

Activated HSC, which was induced by some mediators (TGF-β and PDGF, *etc*.), has been long considered to be associated with liver fibrosis, and inhibition for HSC growth has been proposed as a method for treating liver fibrosis [17,18]. The anti-proliferative activity in PDGF induced HSC-T6 cell was assessed by the MTT assay [19]. Inhibitory activity on cell proliferation was calculated as 100 ° (absorbance of treated compound − absorbance of background light)/(absorbance of model − absorbance of background light). Data were expressed as the mean of the three independent experiments. Colchicine was used as a positive control.

### 3.9. Statistical Analysis

Results were expressed as mean ± SD for at least three analyses for each sample. Statistical analyses were performed using SPSS 12.0 software. The significance of difference was calculated by one-way ANOVA test, and values with *p* < 0.05 were considered to be statistically significant. Graphs were drawn with Microsoft Excel 2003 software.

## 4. Conclusions

In summary, the anti-proliferative activity of *P. chinense* was tested on HSC-T6 cells. In addition, four fractions were also tested their anti-proliferative activity to figure out the active constituents, and six polyphenols were isolated from the EtOAc fraction. The result showed that EtOAc part possesses stronger inhibitory activity than other parts, and the polyphenols may be the active ingredients of *P. chinense* based on the current research. Moreover, two new isolates **1** and **2** have been added to the chemical components of this species. 

## Supplementary Materials

Supplementary materials can be accessed at: http://www.mdpi.com/1420-3049/19/8/11045/s1.
