# Supplementary material for: Polyphenols with Anti-Proliferative Activities from Penthorum Chinense Pursh
Source: Molecules. 2014 Jul 29;19(8):11045–55. doi: 10.3390/molecules190811045 (PMC6271895; doi:10.3390/molecules190811045)

Supplementary Materials

Figure S1. IR of compound **1**.

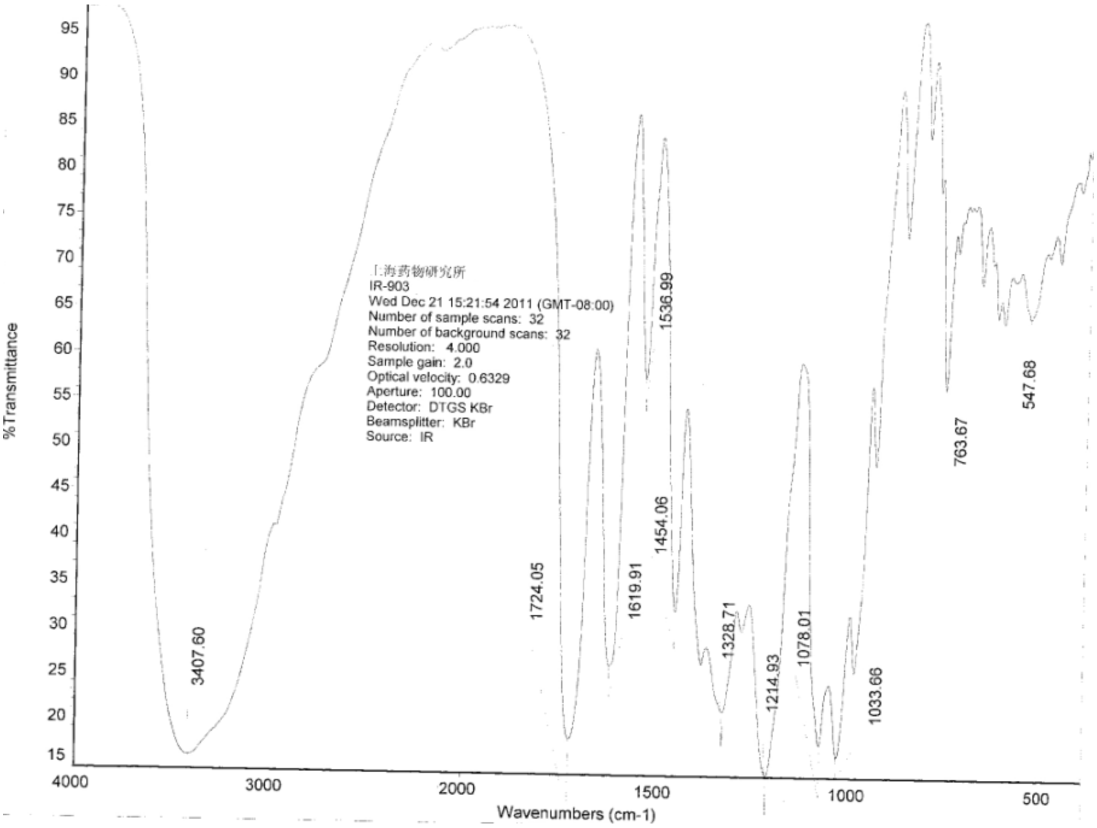

Figure S2. HR-ESI-MS spectrum of compound **1**.

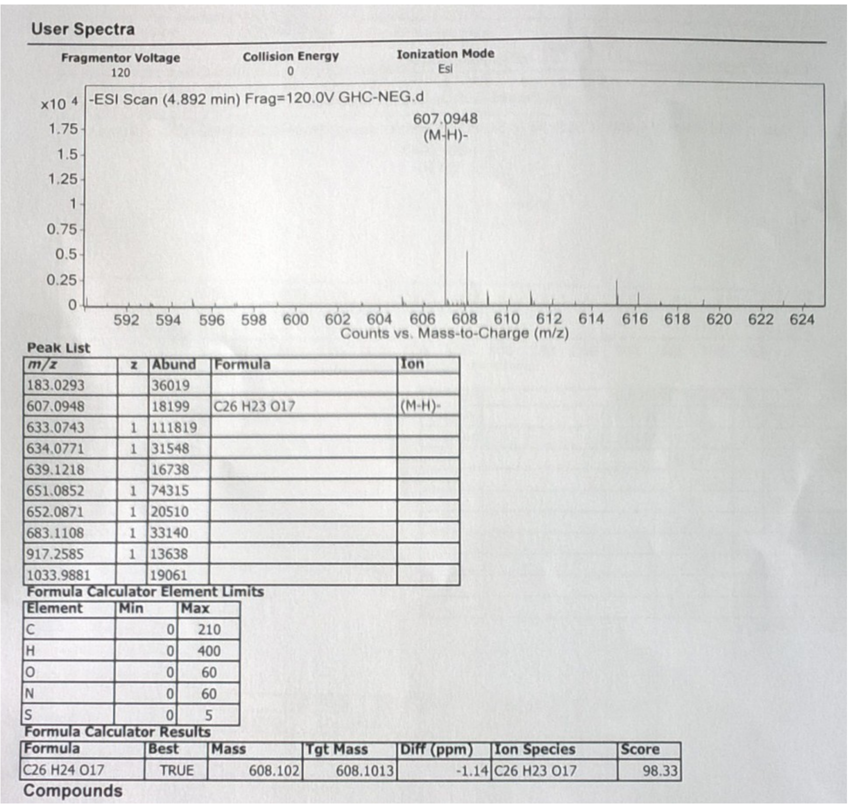

Peak List

m/z	z	Abund	Formula	Ion
183.0293		36019		
607.0948		18199	C26 H23 O17	(M-H)-
633.0743	1	111819		
634.0771	1	31548		
639.1218		16738		
651.0852	1	74315		
652.0871	1	20510		
683.1108	1	33140		
917.2585	1	13638		
1033.9881		19061		

Formula Calculator Element Limits

Element	Min	Max
C	0	210
H	0	400
O	0	60
N	0	60
S	0	5

Formula Calculator Results

Formula	Best	Mass	Tgt Mass	Diff (ppm)	Ion Species	Score
C26 H24 O17	TRUE	608.102	608.1013	-1.14	C26 H23 O17	98.33

Compounds

Figure S3. ^{1}H-NMR (600 MHz, $CDCl_3$) of compound **1**.

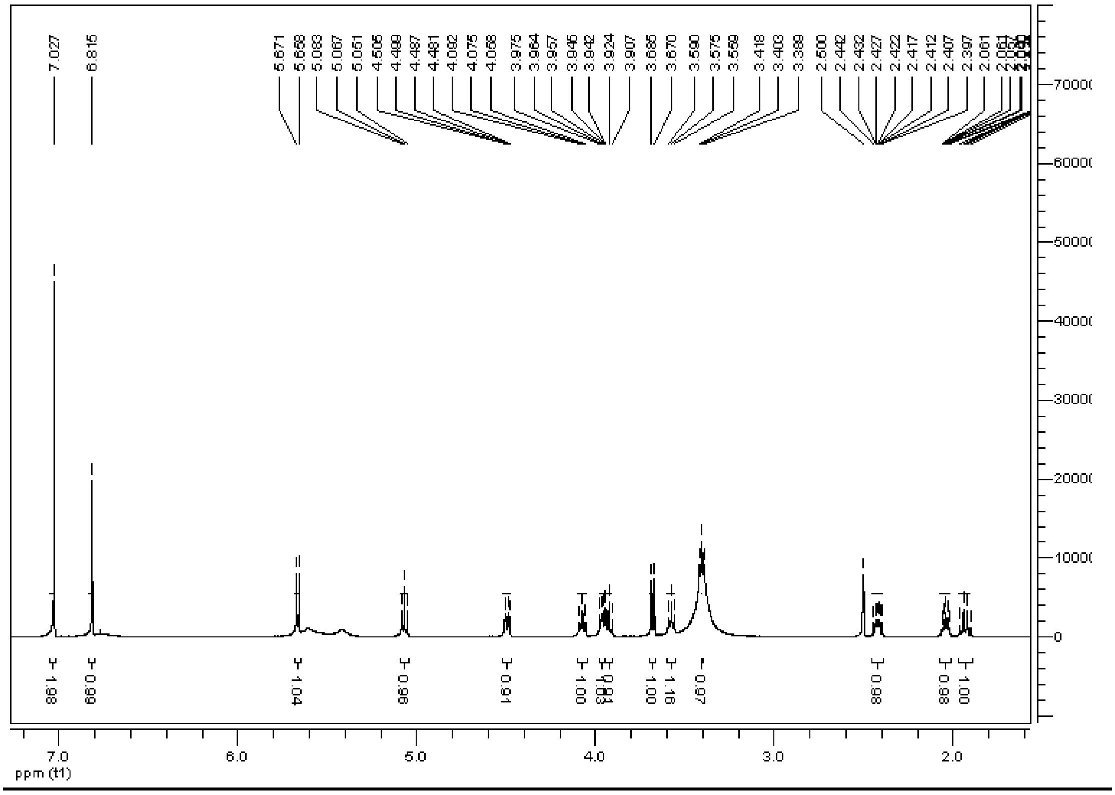

Figure S4. ^{13}C-NMR (150MHz, CDCl3) of compound **1**.

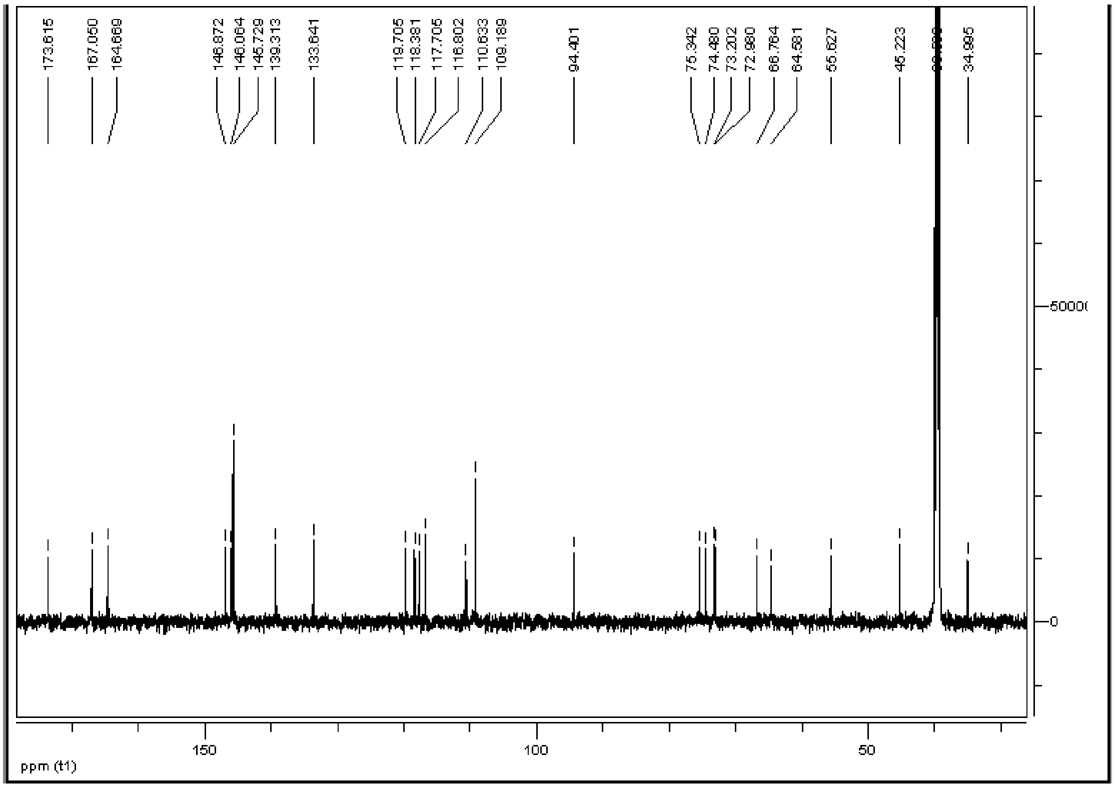

Figure S5. DEPT NMR (150MHz, CDCl3) of compound **1**.

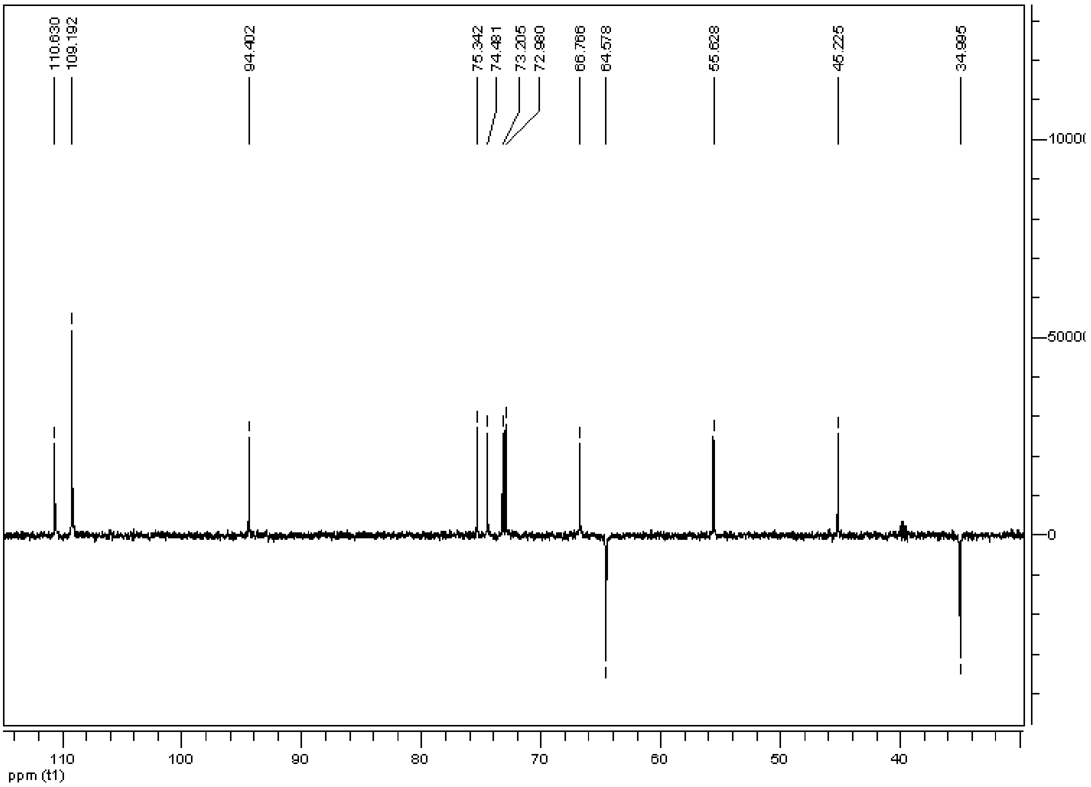

Figure S6. HMQC spectrum of compound **1**.

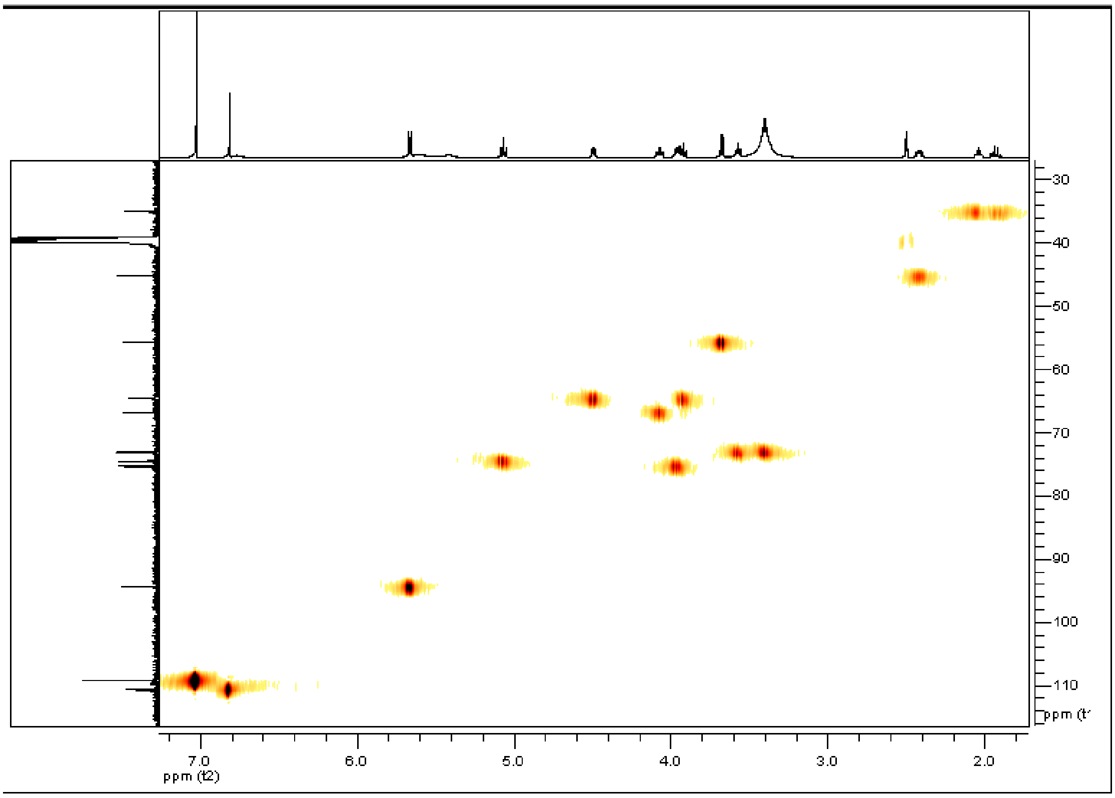

Figure S7. HMBC spectrum of compound **1**.

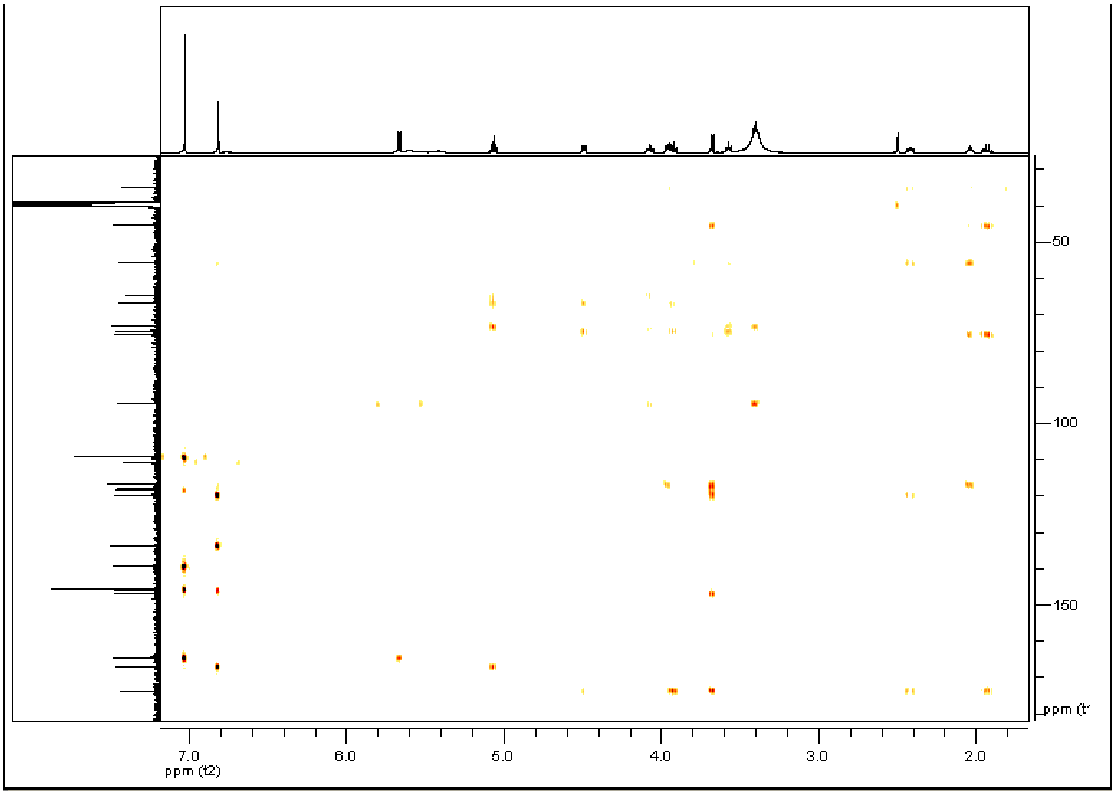

Figure S8. ^{1}H-^{1}H COSY spectrum of compound **1**.

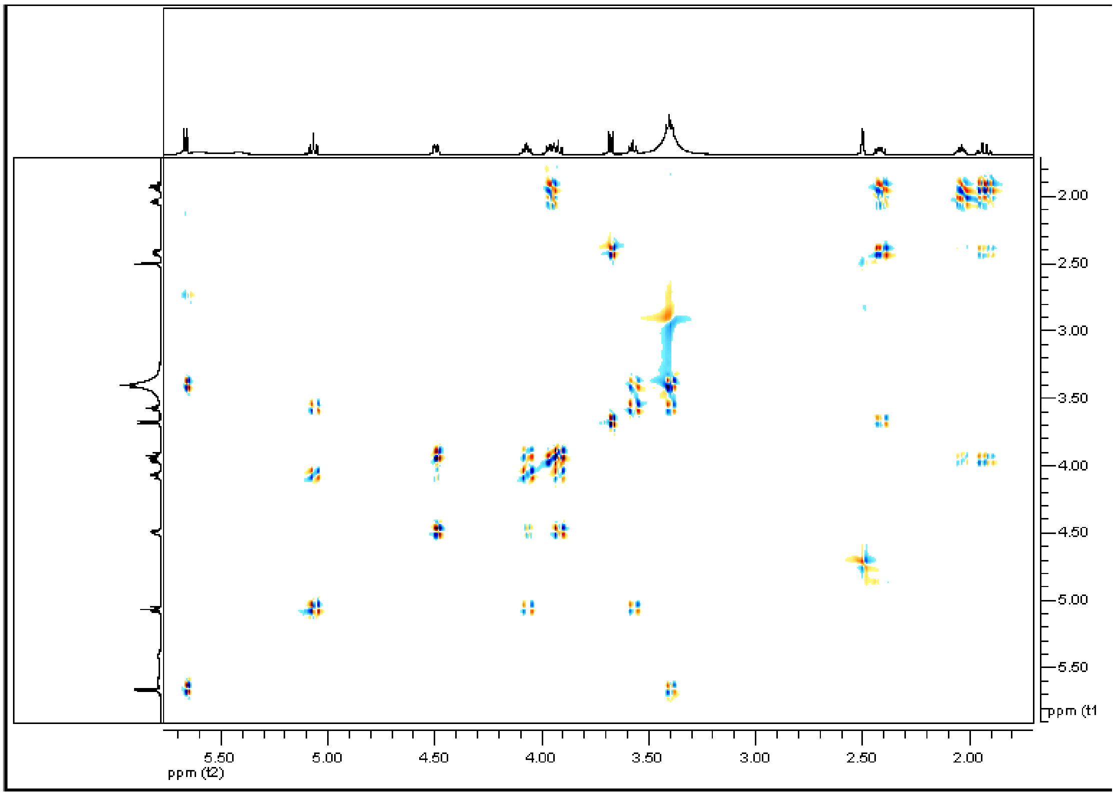

Figure S9. NOESY spectrum of compound **1**.

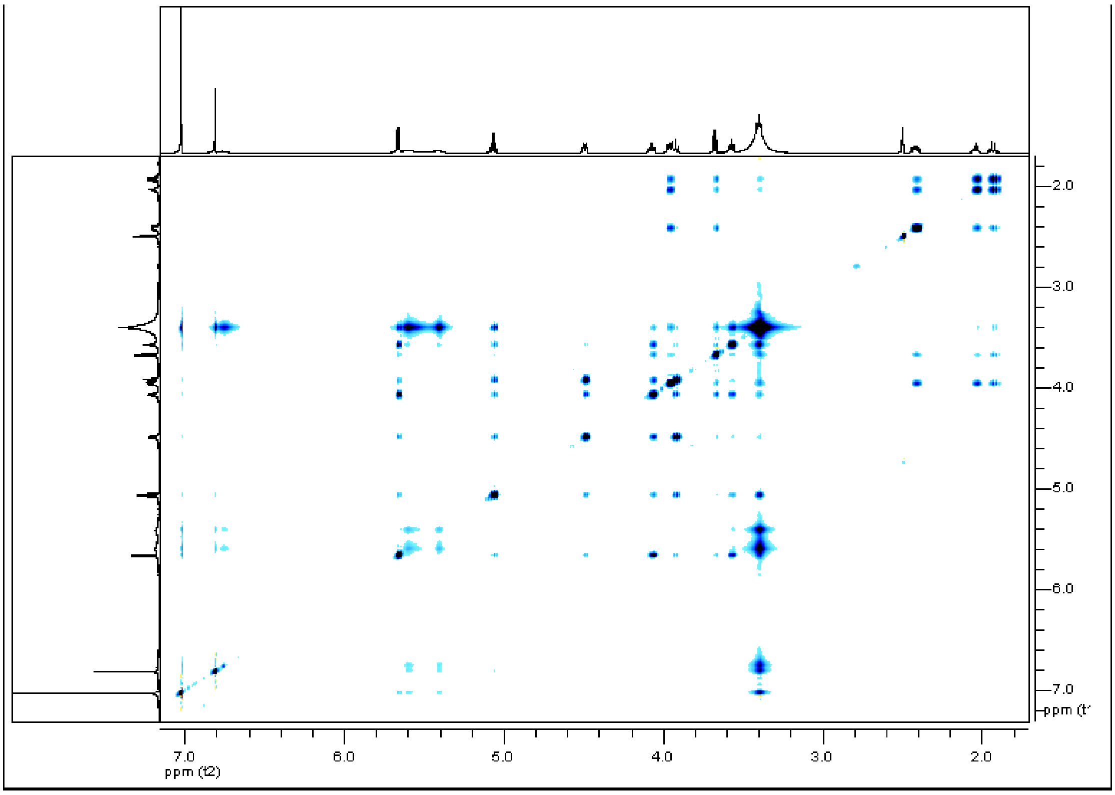

Figure S10. IR of compound **2**.

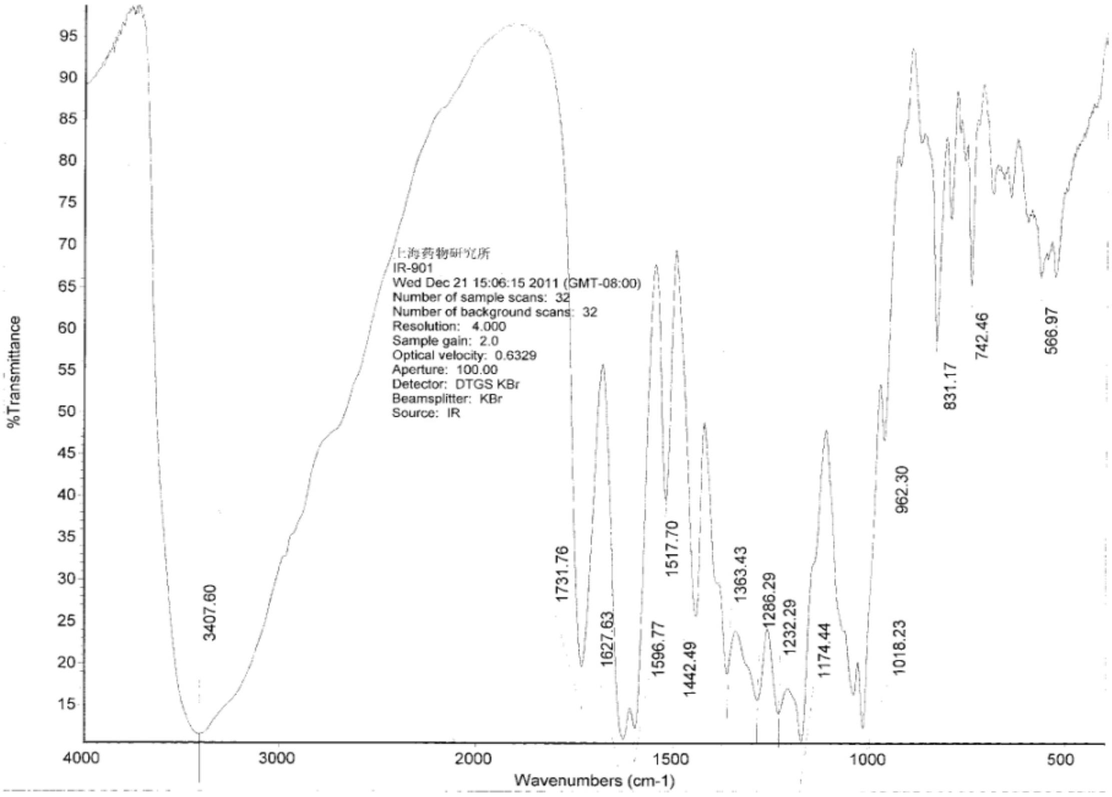

Figure S11. HR-ESI-MS spectrum of compound **2**.

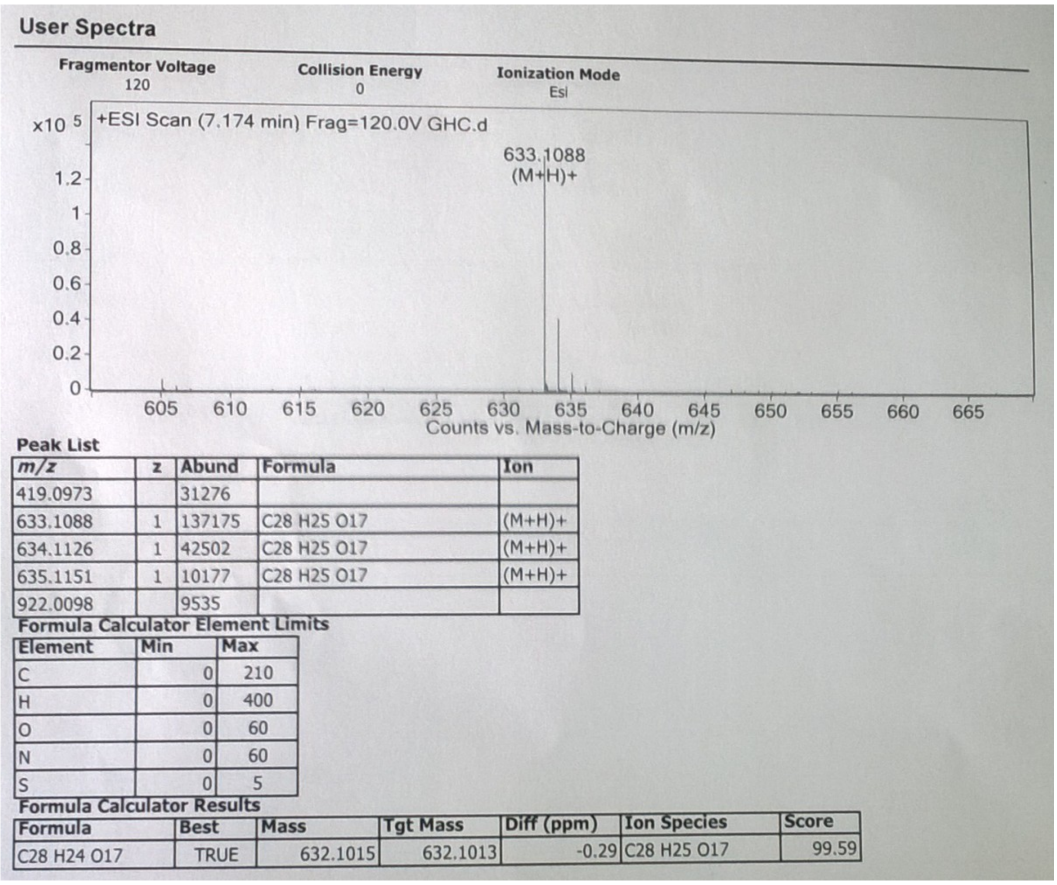

Peak List

m/z	z	Abund	Formula	Ion
419.0973		31276		
633.1088	1	137175	C28 H25 O17	(M+H)+
634.1126	1	42502	C28 H25 O17	(M+H)+
635.1151	1	10177	C28 H25 O17	(M+H)+
922.0098		9535		

Formula Calculator Element Limits

Element	Min	Max
C	0	210
H	0	400
O	0	60
N	0	60
S	0	5

Formula Calculator Results

Formula	Best	Mass	Tgt Mass	Diff (ppm)	Ion Species	Score
C28 H24 O17	TRUE	632.1015	632.1013	-0.29	C28 H25 O17	99.59

Figure S12. ^{1}H-NMR (600 MHz, $CDCl_3$) of compound **2**.

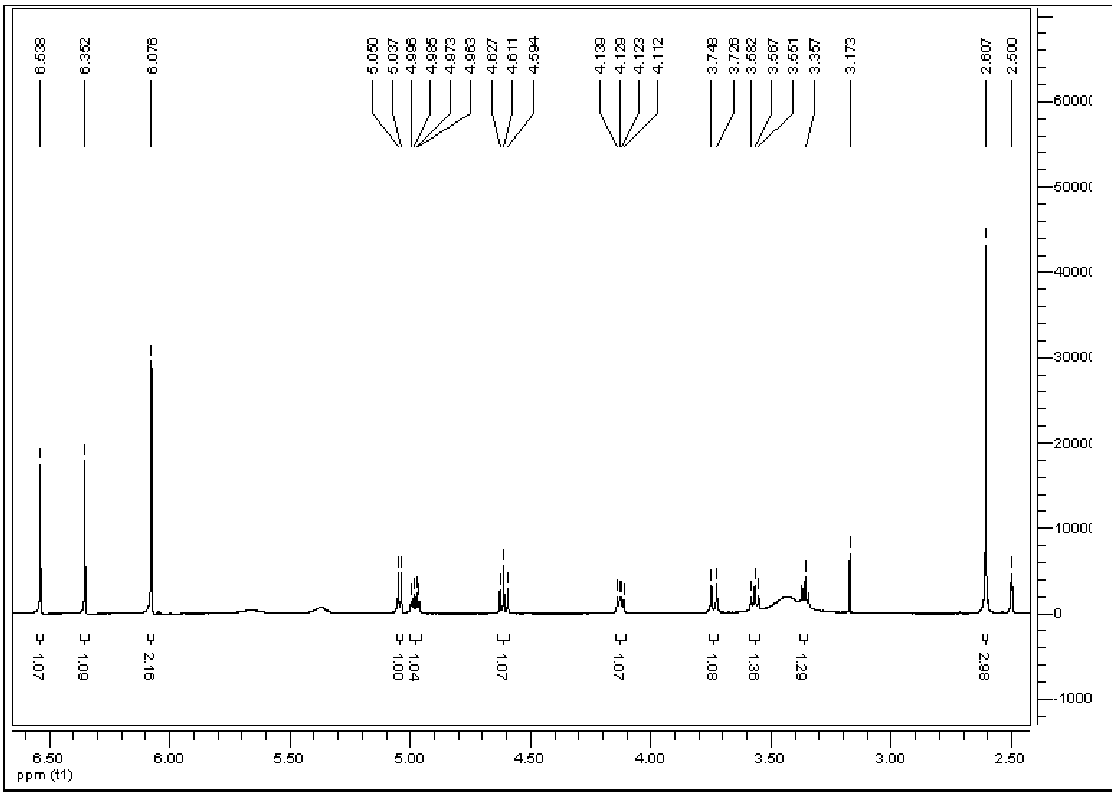

Figure S13. ^{13}C-NMR (150MHz, $CDCl_3$) of compound **2**.

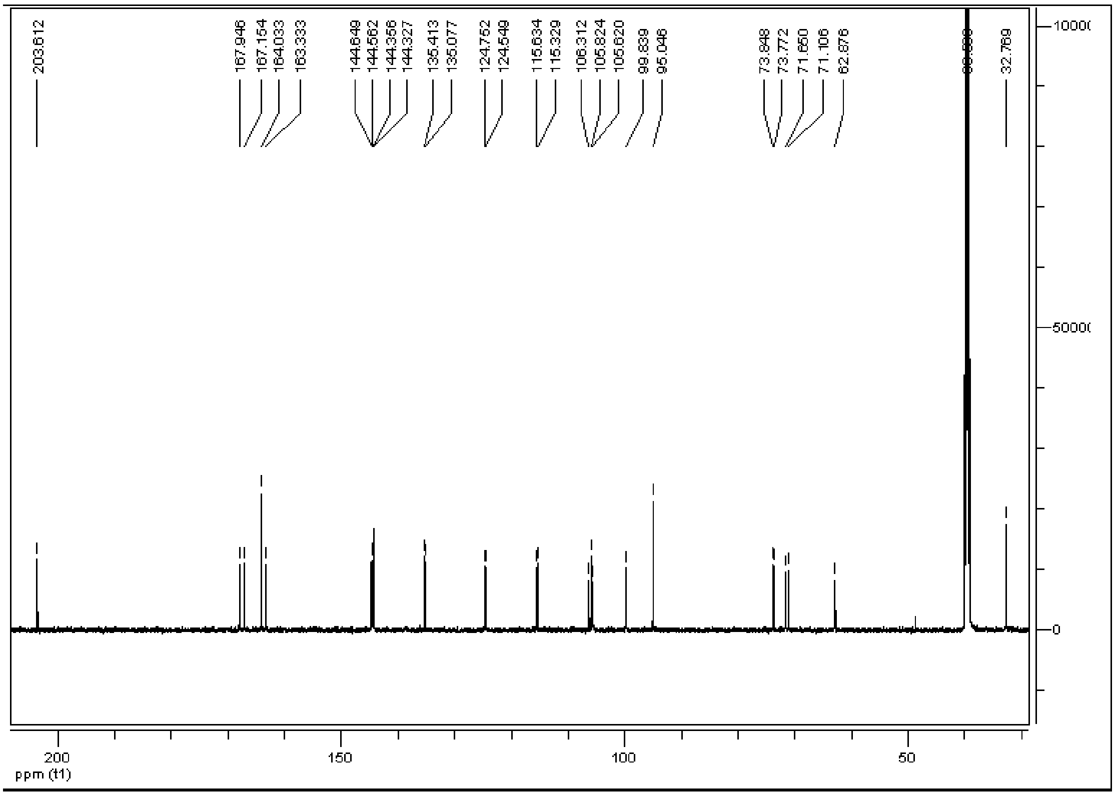

Figure S14. DEPT NMR (150MHz, $CDCl_3$) of compound **2**.

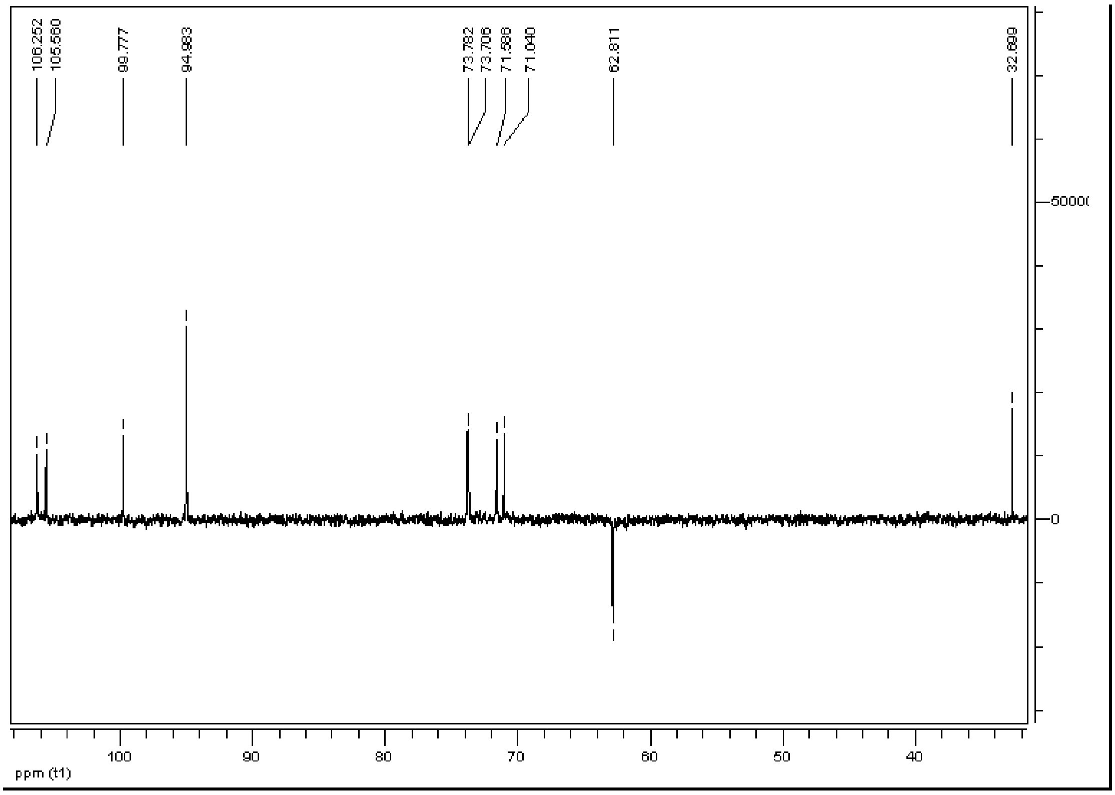

Figure S15. HMQC spectrum of compound **2**.

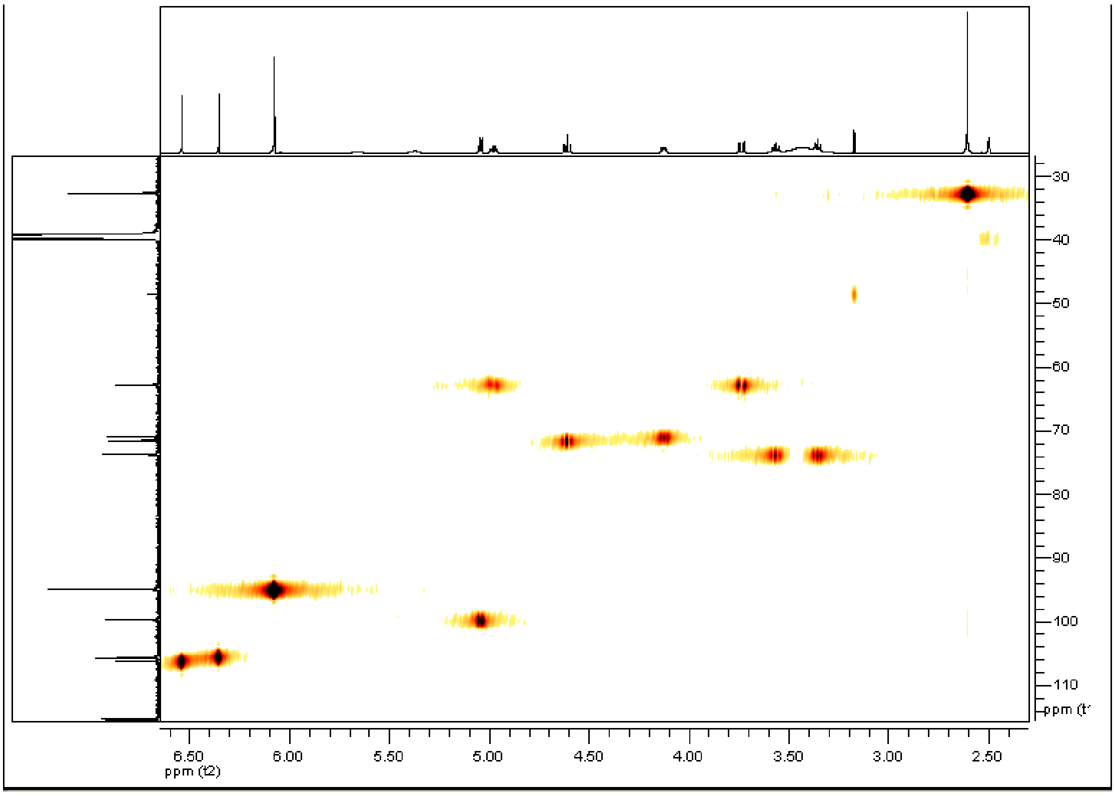

Figure 16. HMBC spectrum of compound **2**.

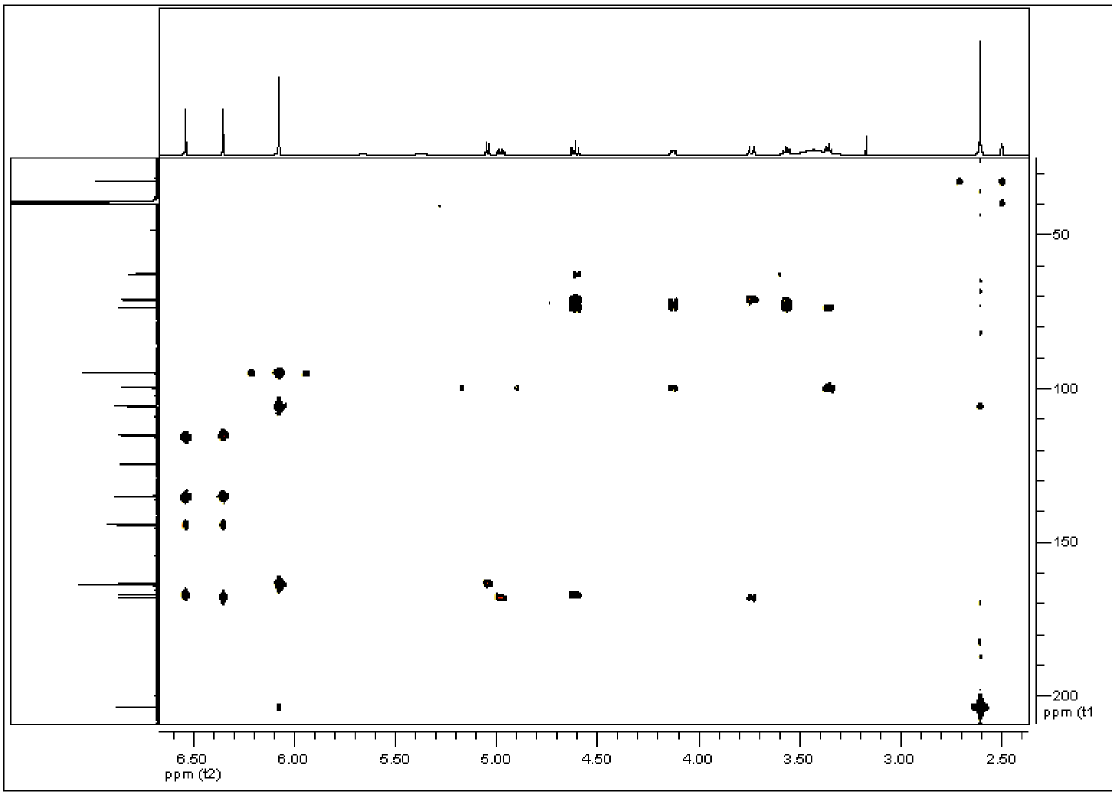

Figure S17. ^{1}H-^{1}H COSY spectrum of compound **2**.

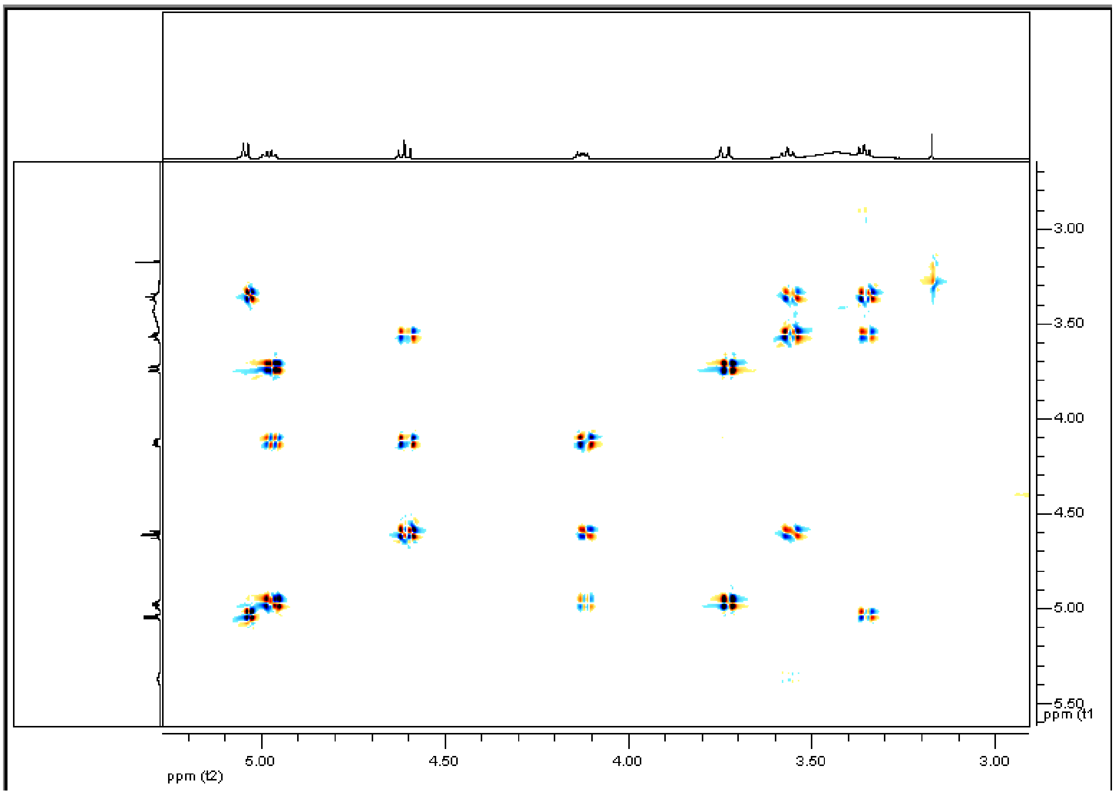

Figure S18. CD spectrum of compound **2**.

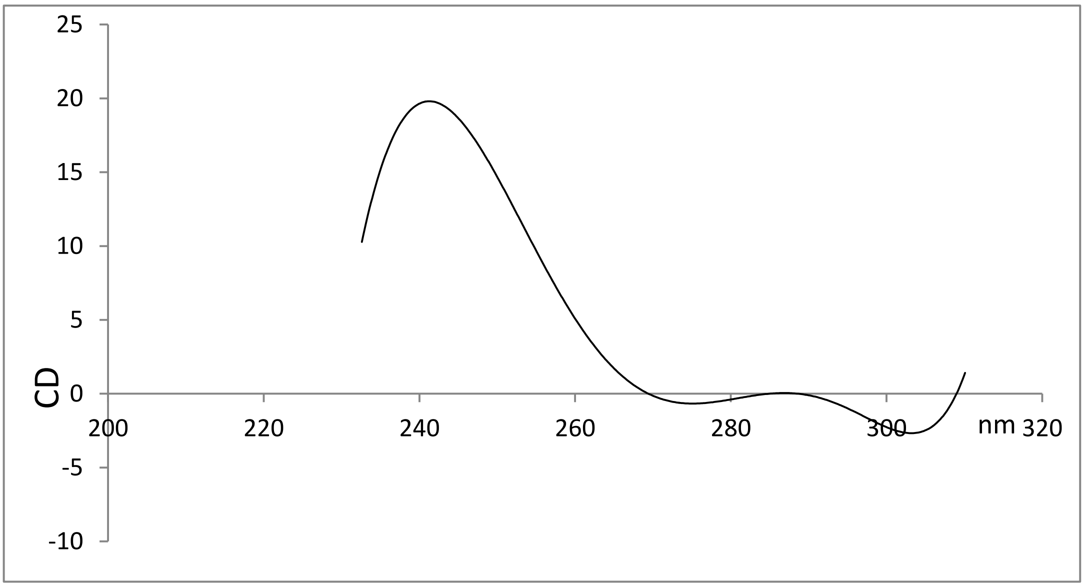

Supplement: Supplementary file 1 [file molecules-19-11045-s001.pdf]
